# Dataset on the use of the Ratkowsky model for describing the influence of storage temperature on microbial growth in hake fillets (*Merluccius merluccius*) stored under MAP

**DOI:** 10.1016/j.dib.2019.104743

**Published:** 2019-11-05

**Authors:** Adriana Antunes-Rohling, Ángela Artaiz, Silvia Calero, Nabil Halaihel, Silvia Guillén, Javier Raso, Ignacio Álvarez, Guillermo Cebrián

**Affiliations:** a Departamento de Producción Animal y Ciencia de los Alimentos, Facultad de Veterinaria, Instituto Agroalimentario de Aragón– IA2, Universidad de Zaragoza-CITA, 50013, Zaragoza, Spain; b Departamento de Patología Animal, Facultad de Veterinaria, Universidad de Zaragoza, 50013, Zaragoza, Spain; c Departamento I+D+i Alquizvetek S.L, Zaragoza, 50013, Zaragoza, Spain

**Keywords:** Shelf-life, Predictive microbiology, Fish, Storage temperature

## Abstract

This article presents the results obtained after applying the Ratkowsky model for developing secondary models describing the influence of storage temperature on microbial growth in hake fillets packaged under a modified atmosphere (MAP) rich in CO_2_ (50% CO_2_/50% N_2_). For this purpose the growth parameters (λ, μ_max_) already calculated in the related article “Modelling microbial growth in Modified-Atmosphere-Packed hake (*Merluccius merluccius*) fillets stored at different temperatures” [1] were used. The data include the fit and goodness of the fit parameters calculated as well as the comparison between fitted and observed data.

**Table:** Specifications Table

Subject area	*Microbiology*
More specific subject area	*Predictive Food Microbiology*
Type of data	*Tables and Figures*
How data was acquired	*Data acquisition: plate counts and qPCR (CFX Connect Real-Time System; Bio-Rad Laboratories, Hercules, USA). Modelization: GraphPad PRISM software (Graph Software, San Diego, CA) and Microsoft Excel software (Microsoft, Seattle, WA).*
Data format	*Raw and analyzed*
Experimental factors	*Influence of growth temperature on growth parameters of different microbial groups*
Experimental features	*Influence of storage temperature on the microbiota of Modified-Atmosphere-Packed (50% CO*_*2*_*/50% N*_*2*_*) hake (Merluccius merluccius) fillets.*
Data source location	*University of Zaragoza, Zaragoza, Spain.*
Data accessibility	*Data are available with this article*
*Related research article*	*Antunes-Rohling, A., Artáiz, A., Calero, S., Halaiher, N., Guillén, S., Raso, J., Álvarez, I., Cebrián, G. Modelling microbial growth in Modified-Atmosphere-Packed hake (Merluccius merluccius) fillets stored at different temperatures. Food Research International, 122, 506–516.* [1] https://doi.org/10.1016/j.foodres.2019.05.018

**Table:** 

**Value of the Data** • The data here presented can be used for estimating the shelf-life of hake stored under MAP at different temperatures. • These data might be useful not only for the fishery industry, but also for food safety authorities, retailers and even consumers. • They can also be used to get further insights into the spoilage process of hake and to better understand the effect of temperature on hake's microbiota. • In contrast to the secondary models described in the related article “Modelling microbial growth in Modified-Atmosphere-Packed hake (Merluccius merluccius) fillets stored at different temperatures” those developed and included in this one are based on the widely used Ratkowsky model. This makes them easier to be implemented in already existing food safety and spoilage prediction programs and/or databases.

## 1 Data

**Fig. 1:**
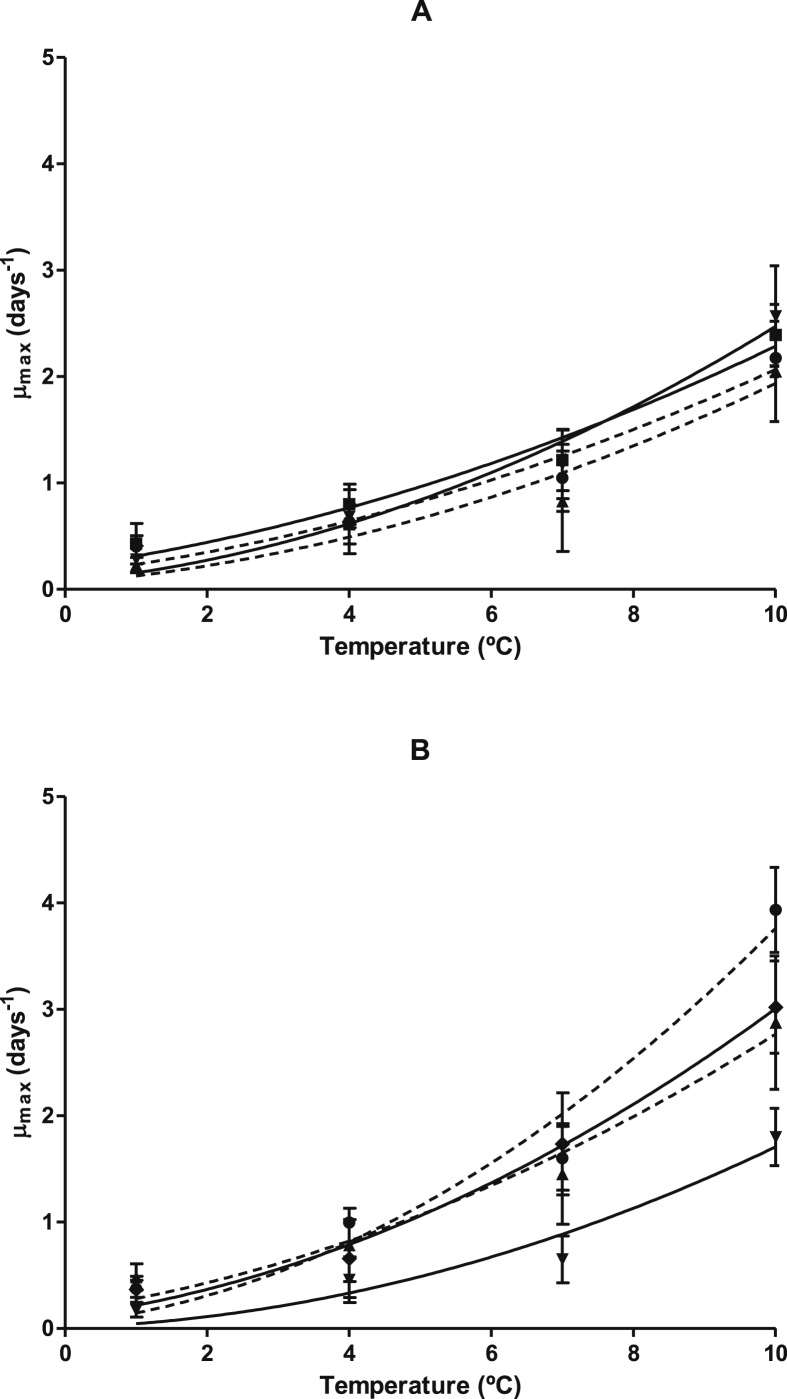
Influence of storage temperature on the μ_max_ values (days-1) of the different microbial groups in hake fillets stored under MAP (50% CO_2_/50% N_2_). A) Non-specific microbial groups: Aerobic Mesophiles (●, discontinuous line), Anaerobic Mesophiles (■, continuous line), Aerobic Psychrotrophes (▲, discontinuous line) and Anaerobic Psychrotrophes (▼, continuous line). B) Specific Microbial groups: *Photobacterium* (●, discontinuous line), *Pseudomonas* (▲, continuous line), *Shewanella* (■, discontinuous line) and Lactic Acid Bacteria (▼, continuous line). Error bars represent the standard error. Lines correspond to the fit to the Ratkowsky model.

**Fig. 2:**
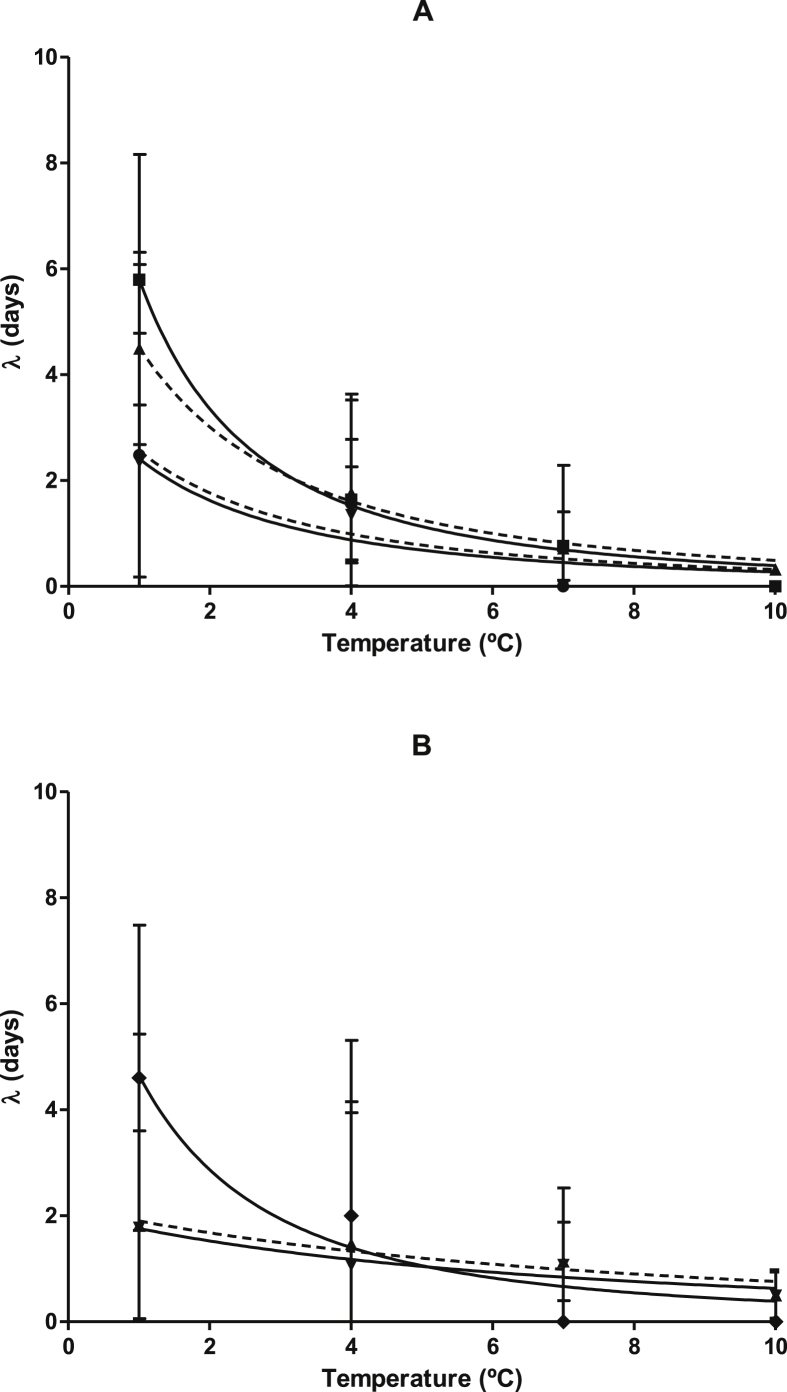
Influence of storage temperature on the λ values (days) of the different microbial groups in hake fillets stored under MAP (50% CO_2_/50% N_2_). A) Non-specific microbial groups: Aerobic Mesophiles (●, discontinuous line), Anaerobic Mesophiles (■, continuous line), Aerobic Psychrotrophes (▲, discontinuous line) and Anaerobic Psychrotrophes (▼, continuous line). B) Specific Microbial groups: *Photobacterium* (●, discontinuous line), *Pseudomonas* (▲, continuous line), *Shewanella* (■, discontinuous line) and Lactic Acid Bacteria (▼, continuous line). Error bars represent the standard error. Lines correspond to the fit to the inverse Ratkowsky model.

**Fig. 3:**
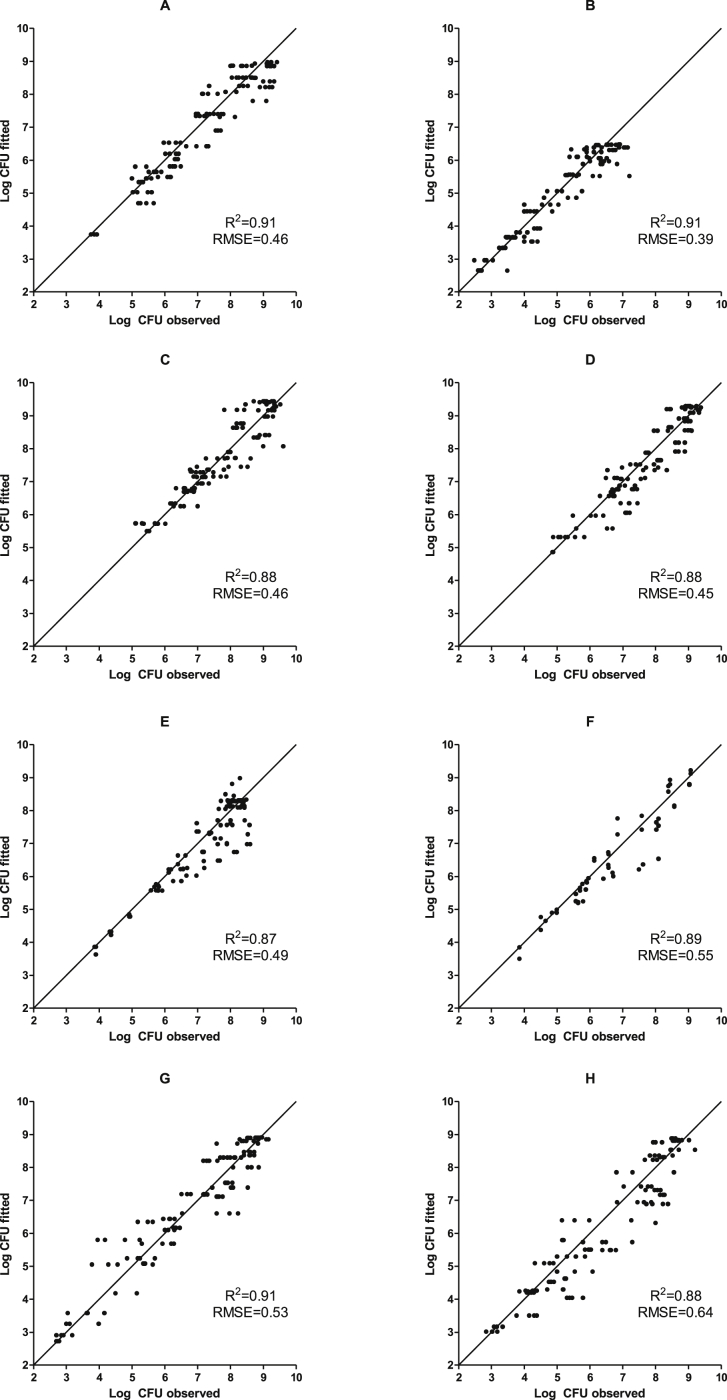
Observed and fitted number of Aerobic Mesophiles (A), Anaerobic Mesophiles (B), Aerobic Psychrotrophes (C), Anaerobic Psychrotrophes (D), *Photobacterium* (E), *Pseudomonas* (F), *Shewanella* (G) and Lactic Acid Bacteria (H). Each figure includes the R^2^ and RMSE values. Data correspond to the 4 temperatures studied and the fitting using the Ratkowsky and inverse Ratkowsky model for μ_max_ and λ, respectively.

Growth curves in hake fillets stored under MAP (50% CO_2_/50% N_2_) of 8 microbial groups were obtained and fitted using the Baranyi and Roberts model [2,3] in the related research article “Modelling microbial growth in Modified-Atmosphere-Packed hake (*Merluccius merluccius*) fillets stored at different temperatures” (Table 1). In this article the Ratkowsky and inverse Ratkowsky model [4,5] are used for describing the influence of storage temperature on the previously calculated growth parameters (*μ*_max_ and *λ*). The influence of storage temperature on the μmax (2) and λ (3) values calculated for each bacterial group (non-specific: 1A and 2A; specific: 1B and 2B) is shown in Fig. 1, Fig. 2 and the values calculated for the fit parameters (*b*, *T*_min_) together with its standard errors are included in Table 2 (storage temperature *vsμ*_*max*_) and Table 3 (storage temperature *vs λ*). The secondary models developed are included in Table 4. Experimentally determined values were compared with those predicted by the models and these results are shown in Fig. 3, which includes data from all the storage temperatures assayed. It also includes the R^2^ and RMSE values calculated for each microbial group.

**Table 1:** Growth and goodness of the fit parameters calculated (Baranyi model) for the different microbial groups in hake fillets stored under MAP (50% CO_2_/50% N_2_) at the 4 different temperatures studied. Adapted from Antunes-Rohling et al., 2019 [1] with permission of Elsevier.

Microbial group	T (° C)	μ_max_ (1/days)		λ (days)		Y_end_ (Log CFU/g)		R^2^	RMSE
		μ_max_	s.e	λ	s.e.	Y_end_	s.e		
Aerobic mesophiles	1	0.40	0.10	2.48	2.31	9.51	0.52	0.98	0.70
	4	0.64	0.30	1.56	2.07	8.25	0.51	0.95	0.63
	7	1.05	0.32	–	–	8.86	0.44	0.96	0.65
	10	2.18	0.18	–	–	9.27	0.13	1.00	0.41
Anaerobic mesophiles	1	0.43	0.19	5.79	2.37	6.22	0.27	0.95	0.60
	4	0.80	0.19	1.64	1.14	6.37	0.19	0.99	0.51
	7	1.22	0.29	0.76	0.65	6.37	0.19	0.99	0.48
	10	2.39	0.29	–	–	6.95	0.12	0.99	0.44
Aerobic psycrotrophes	1	0.23	0.05	4.49	1.82	9.75	0.69	0.99	0.37
	4	0.61	0.18	1.77	1.76	9.68	0.81	0.98	0.56
	7	0.83	0.47	0.72	1.57	9.12	0.39	0.92	0.61
	10	2.05	0.47	0.32	0.10	9.20	0.16	0.99	0.49
Anaerobic psycrotrophes	1	0.28	0.13	2.36	3.73	9.54	0.72	0.96	0.56
	4	0.68	0.10	1.35	0.91	9.50	0.41	0.99	0.43
	7	1.18	0.32	–	–	9.00	0.24	0.97	0.50
	10	2.57	0.47	–	–	9.12	0.13	0.99	0.43
*Photobacterium*	1	0.37	0.08	–	–	8.77	0.30	0.97	0.53
	4	0.99	0.05	–	–	8.16	0.09	1.00	0.32
	7	1.60	0.30	–	–	8.10	0.10	0.98	0.46
	10	3.94	0.40	–	–	8.20	0.70	0.99	0.40
*Pseudomonas*	1	0.17	0.07	1.78	3.65	9.18	1.93	0.98	0.43
	4	0.46	0.22	1.06	3.09	9.05	1.93	0.95	0.63
	7	0.65	0.22	1.08	1.45	9.49	1.40	0.99	0.49
	10	1.80	0.27	0.50	0.48	9.34	0.30	0.99	0.52
*Shewanella*	1	0.48	0.13	1.83	1.77	8.67	0.31	0.98	0.52
	4	0.79	0.34	1.47	2.47	9.53	2.42	0.96	0.79
	7	1.45	0.47	1.14	0.74	8.91	0.24	0.98	0.56
	10	2.88	0.63	0.50	0.43	8.53	0.29	0.99	0.62
Lactic Acid Bacteria	1	0.37	0.12	4.60	2.88	9.42	1.56	0.98	0.61
	4	0.66	0.37	2.00	3.31	9.21	2.74	0.94	0.80
	7	1.74	0.48	–	–	8.30	0.28	0.97	0.65
	10	3.02	0.43	–	–	8.62	0.18	0.99	0.54

(−) No λ was determined.

**Table 2:** Fit (*b*, *Tmin*) and goodness of the fit (R^2^, RMSE) parameters of the Ratkowsky model describing the relationship between μ_max_ and storage temperature.

Microbial Group	*b*	s.e.	*T*_min_	s.e.	R^2^	RMSE
Aerobic Mesophiles	0.11	0.02	−3.58	2.00	0.97	0.15
Anaerobic Mesophiles	0.11	0.02	−4.28	1.76	0.98	0.13
Aerobic Psychrotrophes	0.12	0.02	−2.09	2.20	0.92	0.34
Anaerobic Psychrotrophes	0.13	0.02	−2.00	1.43	0.99	0.14
*Photobacterium*	0.17	0.03	−1.20	1.75	0.98	0.27
*Pseudomonas*	0.12	0.03	−0.75	2.21	0.96	0.16
*Shewanella*	0.13	0.02	−3.20	1.55	0.99	0.15
Lactic Acid Bacteria	0.14	0.01	−2.30	0.88	0.99	0.10

**Table 3:** Fit (*b*, *Tmin*) and goodness of the fit (R^2^, RMSE) parameters of the inverse Ratkowsky model describing the relationship between λ and storage temperature.

Microbial Group	*b*	s.e.	*T*_min_	s.e.	R^2^	RMSE
Aerobic Mesophiles	0.13	0.08	−3.94	3.13	0.85	0.62
Anaerobic Mesophiles	0.13	0.02	−2.17	0.53	0.99	0.29
Aerobic Psychrotrophes	0.10	0.01	−3.76	0.40	0.99	0.14
Anaerobic Psychrotrophes	0.14	0.08	−3.57	0.66	0.99	0.13
*Photobacterium*	–	–	–	–	–	–
*Pseudomonas*	0.06	0.02	−12.4	4.13	0.90	0.20
*Shewanella*	0.05	0.01	−14.5	5.42	0.89	0.24
Lactic Acid Bacteria	0.12	0.06	−2.64	1.66	0.94	0.69

(−) No lag phase was determined at any storage temperature.

**Table 4:** Secondary models developed using for the different microbial groups in hake fillets stored under MAP (50% CO_2_/50% N_2_) at different temperatures (*T*). The models are valid in the range between 1 and 10 °C unless specifically stated.

	μ_max_ model	λ model	Y_end_	
			Mean	s.d.
Aerobic Mesophiles	$\;\sqrt{\mu_{max}}=0.11\;\left(\;T+3.58\right)$	$\frac{1}{\sqrt{\mu_{max}}}=0.13\;\left(\;T+3.94\right)$	8.97	0.55
Anaerobic Mesophiles	$\sqrt{\mu_{max}}=0.11\;\left(\;T+4.28\right)$	$\frac{1}{\sqrt{\mu_{max}}}=0.13\;\left(\;T+2.17\right)$	6.48	0.32
Aerobic Psychrotrophes	$\sqrt{\mu_{max}}=0.12\;\left(\;T+2.09\right)$	$\frac{1}{\sqrt{\mu_{max}}}=0.10\;\left(\;T+3.76\right)$	9.44	0.32
Anaerobic Psychrotrophes	$\sqrt{\mu_{max}}=0.13\;\left(\;T+2.00\right)$	$\frac{1}{\sqrt{\mu_{max}}}=0.14\;\left(\;T+3.57\right)$	9.29	0.27
*Photobacterium*	$\sqrt{\mu_{max}}=0.17\;\left(\;T+1.20\right)$	–	8.31	0.31
*Pseudomonas*	$\sqrt{\mu_{max}}=0.12\;\left(\;T+0.75\right)$	$\frac{1}{\sqrt{\mu_{max}}}=0.06\;\left(\;T+12.4\right)$	9.27	0.19
*Shewanella*	$\sqrt{\mu_{max}}=0.13\;\left(\;T+3.20\right)$	$\frac{1}{\sqrt{\mu_{max}}}=0.05\;\left(\;T+14.5\right)$	8.91	0.44
Lactic Acid Bacteria	$\sqrt{\mu_{max}}=0.14\;\left(\;T+2.30\right)$	$\frac{1}{\sqrt{\mu_{max}}}=0.12\;\left(\;T+2.64\right)$	8.89	0.52

## 2 Experimental design, materials and methods

### 2.1 Development of secondary models and statistical analysis

The growth parameters (Baranyi model [2,3]) previously calculated [1] for 8 bacterial groups (see Table 1) in hake fillets packaged in a modified atmosphere (50% CO_2_/50% N_2_) and stored at four different temperatures (1, 4, 7 & 10 °C) were modelized using the Ratwosky [4] and inverse Ratkowsky model [5].

The Ratkowsky model [4] was used for describing the influence of storage temperature on the *μ*_max_. This model is defined by the following equation:

(1) $$\sqrt{\mu_{max}}=\;b\left(T-T_{0}\right)$$

Where $\sqrt{\mu_{max}}$ is the square root of maximum growth rate, *b* is the slope of the regression line, *T* is temperature, and *T*_*0*_ is a conceptual minimum temperature for microbial growth, where T and *T*_*0*_ are given in °C.

Three influence of storage temperature on lag time (*λ*) was described with the inverse Ratkowsky model [5]:

(2) $$\frac{1}{\sqrt{\lambda }}=\;b\left(T-T_{0}\right)$$

Where λ is the lag time, *b* is the slope of the regression line, *T* is the temperature, and *T*_*0*_ is a conceptual minimum temperature for microbial growth, where T and *T*_*0*_ are given in °C.

GraphPad PRISM software (Graph Software, San Diego, CA) was used for curve fitting, and Microsoft Excel software (Microsoft, Seattle, WA) was used to calculate the goodness of the fit parameters (R^2^, RMSE).
